# Risk SNP-induced lncRNA-SLCC1 drives colorectal cancer through activating glycolysis signaling

**DOI:** 10.1038/s41392-020-00446-7

**Published:** 2021-02-19

**Authors:** Tingting Yan, Chaoqin Shen, Penglei Jiang, Chenyang Yu, Fangfang Guo, Xianglong Tian, Xiaoqiang Zhu, Shiyuan Lu, Bingshe Han, Ming Zhong, Jinxian Chen, Qiang Liu, Yingxuan Chen, Junfang Zhang, Jie Hong, Haoyan Chen, Jing-Yuan Fang

**Affiliations:** 1 State Key Laboratory for Oncogenes and Related Genes; Key Laboratory of Gastroenterology & Hepatology, Ministry of Health; Division of Gastroenterology and Hepatology; Shanghai Cancer Institute; Shanghai Institute of Digestive Disease; Renji Hospital, Shanghai Jiao Tong University School of Medicine, 145 Middle Shandong Road, 200001 Shanghai, China; 2 Key Laboratory of Exploration and Utilization of Aquatic Genetic Resources, Ministry of Education, College of Fishery and Life Science, Shanghai Ocean University, Shanghai, 201306 China; 3 Division of Gastrointestinal Surgery, Renji Hospital, School of Medicine, Shanghai Jiao Tong University, 145 Middle Shandong Road, 200001 Shanghai, China; 4 Department of Pathology, Renji Hospital, School of Medicine, Shanghai Jiao Tong University, 145 Middle Shandong Road, 200001 Shanghai, China

**Keywords:** Gastrointestinal cancer, Epigenetics, Cancer metabolism, Cancer genetics

## Abstract

Long non-coding RNAs (lncRNAs) play key roles in colorectal carcinogenesis. Here, we aimed to identify the risk SNP-induced lncRNAs and to investigate their roles in colorectal carcinogenesis. First, we identified rs6695584 as the causative SNP in 1q41 locus. The A>G mutation of rs6695584 created a protein-binding motif of BATF, altered the enhancer activity, and subsequently activated lncSLCC1 expression. Further validation in two independent CRC cohorts confirmed the upregulation of lncSLCC1 in CRC tissues, and revealed that increased lncSLCC1 expression was associated with poor survival in CRC patients. Mechanistically, lncRNA-SLCC1 interacted with AHR and transcriptionally activated HK2 expression, the crucial enzyme in glucose metabolism, thereby driving the glycolysis pathway and accelerating CRC tumor growth. The functional assays revealed that lncSLCC1 induced glycolysis activation and tumor growth in CRC mediated by HK2. In addition, HK2 was upregulated in colorectal cancer tissues and positively correlated with lncSLCC1 expression and patient survival. Taken together, our findings reveal a risk SNP-mediated oncogene lncRNA-SLCC1 promotes CRC through activating the glycolysis pathway.

## Introduction

Colorectal cancer (CRC) is the second leading cause of cancer-related deaths globally. More than 1.8 million new cases and 881,000 deaths are estimated to occur in the world in 2018.^1^ Although the incidence and mortality in old people have been decreased over the past 30 years, CRC remains a challengeable issue among patients younger than 50 years old.^2,3^ Various studies have explored the genetic and epigenetic biomarkers for CRC risk and the individual’s response to treatment.^4,5^ However, the underlying pathogenesis of CRC is still not fully understood, in which case more effective biomarkers and therapeutic targets are needed to improve the diagnosis and treatment of CRC.

Several risk factors have been proven to contribute to the initiation and development of CRC, among which the genetic heritability of CRC has been estimated at 12–35%.^6,7^ Over the last decade, the genome-wide association studies (GWAS) have provided much information for genetic predisposition to CRC, and have discovered over 50 independent single-nucleotide polymorphisms (SNPs) associated with CRC in both European and Asian populations.^8^ However, most of the loci are located in non-coding regions; thus, the biological functions of these risk SNPs remain poorly understood. Recently, several studies revealed that the regions harboring CRC-associated SNPs might function as a transcriptional enhancer, and interact with transcriptional factors to regulate the expression of target genes.^9,10^ A meta-analysis of three GWAS identified the tag SNP rs6687758, located in non-coding regions of chromosome 1q41, as being significantly associated with CRC, with the G allele conferring the increased risk; however, the causative SNP and the underlying mechanism had yet been explored.^11^ Filling the gap between those risk SNPs and the disease-associated phenotype will be important in understanding the pathogenesis of CRC and facilitating new approaches for prevention and treatment.^12^

Increasing evidence reveal that long non-coding RNAs (lncRNAs) play an important role in various biological processes, including regulation of gene expression, glucose metabolism,^13,14^ maintenance of pluripotency, and differentiation of embryonic stem cells.^15^ Recent studies also demonstrate that lncRNAs are associated with the progression of types of cancers, including CRC.^16^ Furthermore, risk-related SNP rs11672691 can remotely modulate the expression of lncRNA PCAT19 gene, then promoting the progression of aggressive prostate cancer.^17^ Thus, we assumed that rs6687758, located in non-coding regions of chromosome 1q41, might also involve in CRC development via dysregulation of lncRNAs.

The abnormal activation of glycolysis and lactic acid accumulation in the microenvironment have been associated with poor prognosis in CRC patients, which increases malignant features, including cancer proliferation and metastasis.^18,19^ In the current study, we first identify rs6695584 as the causative SNP that participates in CRC progression and demonstrate that the risk allele at rs6695584 is associated with poor prognosis. Then, we determine that rs6695584 contributes to CRC progression by promoting the expression of lncRNA-SLCC1 (previously named RP11-400N13.2) through functioning as an enhancer. In addition, we find that the lncSLCC1 expression upregulated in CRC tissues and increased lncSLCC1 level is associated with poor survival. The mechanistic studies further reveal that lncSLCC1 promotes CRC development through activating HK2 expression by interacting with AHR, finally enhancing glycolysis pathway. Our study reveal a risk SNP-induced lncSLCC1 in glycolysis signaling via HK2 in CRC, suggesting that lncSLCC1 could be a potential new therapeutic target for CRC.

## Results

### The causal SNP rs6695584 is associated with CRC survival and regulates lncSLCC1 expression as an enhancer

**Fig. 1:**
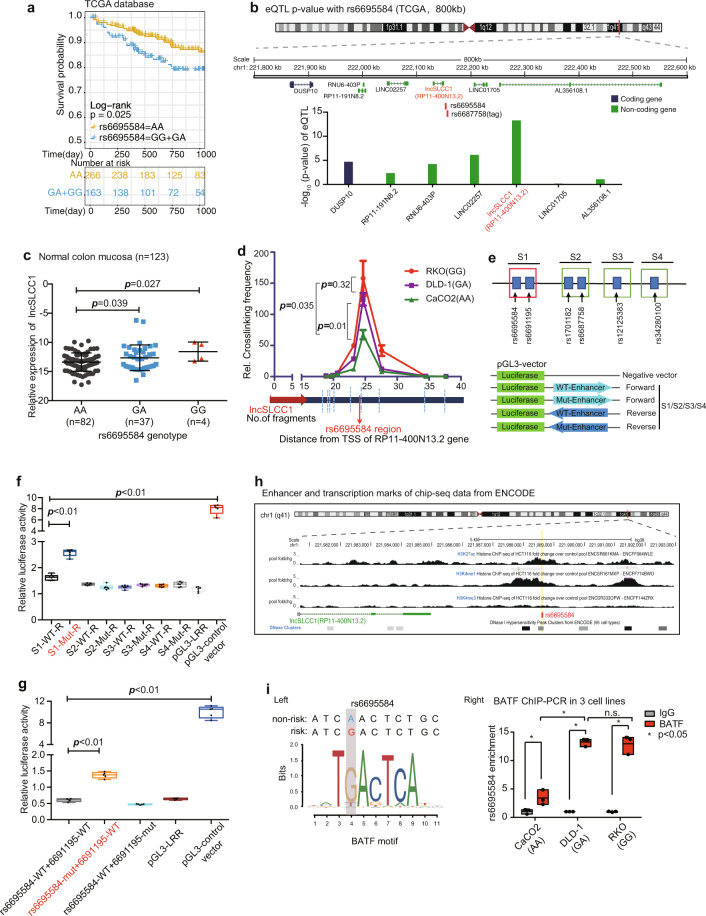
CRC-associated risk SNP rs6695584 regulates lncSLCC1 expression as an enhancer. **a** Kaplan–Meier curve showing the survival probability (3 years) of CRC patients with different genotypes of SNP rs6695584 in TCGA database. **b** rs6695584 eQTL analysis of genes within 800 kb window using data from the TCGA-CRC patients. **c** The mRNA expression of lncSLCC1 in normal colon mucosa with different genotypes of SNP rs6695584 in Cohort 1. **d** 3C-qPCR was performed to determine the relative interaction frequencies between RL (containing SNP rs6695584) and lncSLCC1 promoter region, comparing the relative abundance of ligation products formed between the fragment mapping to RL and each of the target fragments in lncSLCC1 promoter region. **e–g** Relative reporter gene activity of the constructs containing the WT or Mut allele of different SNPs in reverse orientation in DLD-1 cells. **h** Epigenetic tracks obtained from ENCODE database show the enrichment of enhancer marks (H3K27ac and H3K4me1 peaks) and transcription marks (H3K4me3 peaks) in the rs6695584 region. **i** The risk allele G of rs6695584 creates a BATF-binding motif. Left: predicted preferential binding of BATF to the risk allele G of rs6695584. Right: ChIP-qPCR of BATF in CRC cells carrying different rs6695584 genotypes (Caco2[AA], DLD-1[GA], and RKO[GG])

As reported, rs6687758 has been proven to be a risk lead SNP associated with CRC in a large meta-analysis based on GWAS studies.^11^ To further confirm its role in CRC progression, we examined the overall survival of CRC patients based on the rs6687758 genotype in TCGA-CRC cohort (Fig. 1a). Considering that rs6687758 was not present in Affymetrix Genome-Wide Human SNP Array 6.0 of TCGA samples, we took rs6695584 as proxy SNP, which is in strong Linkage disequilibrium (LD) (*r*^2^ = 1, from HaploReg v4.0) with the tag SNP rs6687758. The results showed that patients with G allele (GG+GA) were associated with shorter 3-year overall survival than those with AA genotype, which revealed the role of the risk SNP rs6695584 in the progression of CRC.

The expression quantitative trait loci (eQTL) analysis could provide immediate insight into a biological basis for disease association identified through GWAS studies.^20^ To elucidate the causative variant of 1q41, we performed eQTL analysis in an 800k base-pair window in the TCGA-CRC cohort (Fig. 1b). rs6695584 was located between lncSLCC1 and LINC01705 in chr1, and seven candidate genes were found to be putative targets of the risk SNP. Among all the genes, *cis*-eQTL revealed the strongest association between rs6695584 and lncSLCC1 (Fig. 1b). Consistent with the eQTL data, CRC samples with risk allele genotype G (GG or GA) had higher expression of lncSLCC1 compared to those with non-risk allele genotype A (Supplementary Fig. S1a). Further supporting data showed that normal colon mucosa samples with GG or GA genotype of rs6695584 had significantly higher expression of lncSLCC1 than AA genotype in cohort 1 (*n* = 123, Fig. 1c). Besides, rs6695584 showed evidence of association with CRC in Singaporean Chinese.^21^ Taken together, rs6695584 might contribute to CRC progression by regulating the expression of lncSLCC1.

Given that the association between rs6695584 and the expression of lncSLCC1 was confirmed, the underlying mechanism was unclear. To evaluate whether there was a direct chromatin interaction between the region of rs6695584 and lncSLCC1 promoter, we examined the genotype of three common CRC cell lines (Caco2, DLD-1, and RKO) (Supplementary Fig. S1b, c) and performed chromosome conformation capture (3C) assay. When anchored at lncSLCC1 promoter, the rs6695584 region showed a strong interaction with lncSLCC1 promoter, and the interaction was enriched in RKO and DLD-1 cells with the risk allele of rs6695584 (Fig. 1d).

Functional SNPs are believed to modify the activity of transcriptional regulatory regions.^12^ To evaluate whether rs6695584 region function as an enhancer and regulate lncSLCC1 expression, the risk SNP region between 10 kb upstream and downstream of lead SNP rs6687758 was divided into four segments, which were named S1 (containing rs6695584 and rs6691195), S2 (containing rs17011182 and rs6687758), S3 (containing rs12125383), and S4 (containing rs34280100) (Fig. 1e). Then, we performed luciferase-based enhancer assays by cloning the four segments (wild type and mutated) into the reporter vectors. The results showed that only S1-Reverse had significantly higher activity than the control vector and other segments (Fig. 1f and Supplementary Fig. S1d), suggesting that S1 was the functional part of the risk SNP region as an enhancer. To identify the specific causative SNP located in S1, the two SNPs were next individually mutated and significantly increased luciferase activity was only observed in vectors with a risk allele G of rs6695584 (Fig. 1g), implying that rs6695584 was the causative SNP. Moreover, the luciferase result was further supported by H3K27ac, H3K4me1, and H3K4me3 (transcription marker, not enrichment) ChIP-seq data from the ENCODE project (Fig. 1h). Besides, we performed the Polymerase II ChIP-PCR, the result showed that Polymerase II occupancy on the rs6695584 region (Supplementary Fig. S1e).

Given that SNP-specific changes are thought to modify enhancer activity by altering transcription factor (TF) binding,^22^ we next examined whether rs6695584 directly alters the DNA-binding motif by using FIMO and JASPAR. This analysis indicated that rs6695584 overlaps with the binding motif of transcriptional factor BATF (Fig. 1i). Notably, BATF has a higher preference for the risk allele “G” (Fig.1i, left). In line with the motif analysis, ChIP-PCR also showed that a stronger BATF binding is enriched in the rs6695584 region in DLD-1(GA) and RKO(GG) cells than CaCO2 cells(AA) (Fig. 1i, right). Altogether, we demonstrated that rs6695584 was a risk SNP and contributed to CRC progression by promoting the expression of lncSLCC1 through functioning as an enhancer. Therefore, we focused our research on lncRNA-SLCC1 (short for “SNP-associated lncRNA of CRC”).

### LncRNA-SLCC1 is clinically relevant in CRC

We measured the expression of lncSLCC1 in different CRC cell lines. As shown in Supplementary Fig. S1c, DLD-1 cell line has a relatively high expression level of lncSLCC1, and HT29 exhibits relatively low-expression level of lncSLCC1. So, we performed loss-of function assay in DLD-1 cells and gain-of function assay in HT29 cells. Firstly, to define whether lncSLCC1 is a novel lncRNA, we performed the 5′ and 3′ rapid amplification of cDNA ends PCR (Supplementary Fig. S2a–c). Furthermore, we confirmed that lncSLCC1 was unlikely to encode any protein product by in vitro translation analysis (Supplementary Fig. S2d). Also, we found that lncSLCC1 was mainly located in the nuclear fraction (Supplementary Fig. S2e). Collectively, lncSLCC1 is a novel lncRNA whose expression is positively correlated with the risk genotype of SNP rs6695584 in CRC cells.

**Fig. 2:**
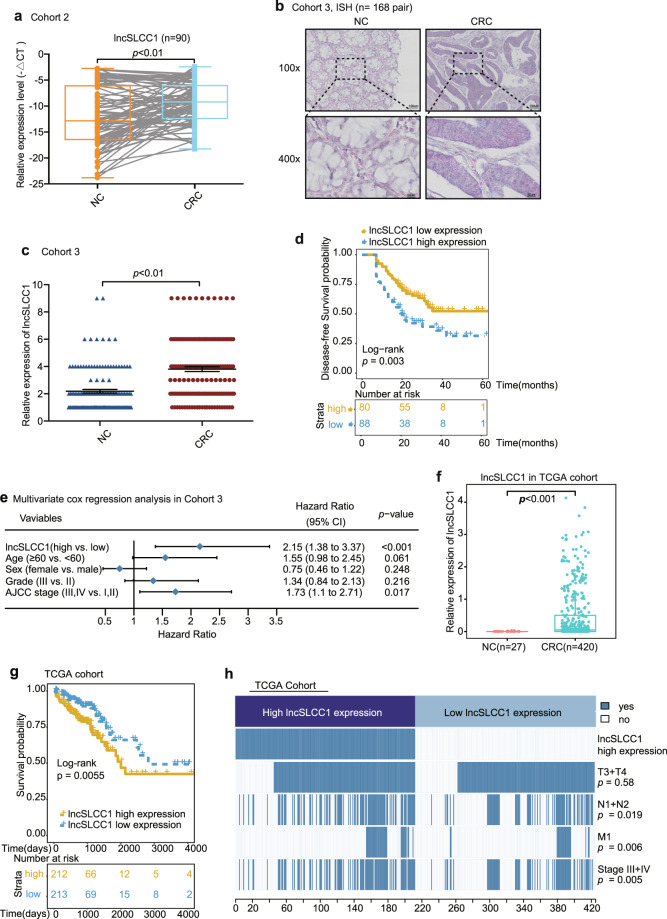
LncRNA-SLCC1 is clinically relevant in colorectal cancer. **a** LncSLCC1 mRNA expression in 90 pairs of colorectal cancer and normal tissues in Cohort 2, paired *t-*test. **b** Representative images of lncSLCC1 expression in colorectal cancer and adjacent colorectal tissues using ISH analysis in Cohort 3; *n* = 168. **c** Summarized data of lncSLCC1 expression in Cohort 3; *n* = 168. **d** The overall survival rate was analyzed and compared between patients with high and low levels of lncSLCC1 expression in tumor in Cohort 3; *n* = 168, log-rank test. **e** Multivariate cox regression analysis of lncSLCC1 in Cohort 3. **f** LncSLCC1 mRNA expression in colorectal cancer and adjacent colorectal tissues in TCGA dataset. **g** Kaplan–Meier curves showing the survival rate of patients with different levels of lncSLCC1 in TCGA dataset. **h** Comparing different TNM and AJCC stage between lncSLCC1 high- and low-expression tumors in TCGA dataset. The heatmap illustrates the association of different clinical characters with lncSLCC1 high- and low-expression tumors. Statistical significance was performed by the *χ*^2^ test

Next, we revealed that lncSLCC1 expression was significantly increased in CRC cancer tissues compared to adjacent normal tissues of CRC patients in Cohort 2 (Fig. 2a, fold change = 7.01). To further confirm the results, we detected and compared lncSLCC1 expression by in situ hybridization in an additional cohort containing 168 paraffin-embedded CRC and adjacent tissues (Cohort 3, Supplementary Table S1). Consistent with the results in Cohort 2, lncSLCC1 expression was significantly higher in CRC tissues than in adjacent tissues (Fig. 2b, c, fold change = 1.74, Supplementary Fig. S2f). Clinically, the Kaplan–Meier analyses showed that high expression of lncRNA-SLCC1 was significantly associated with a poor prognosis in these patients (Fig. 2d). The multivariate regression analyses further demonstrated that lncSLCC1 expression was an independent predictor of CRC. Its predictive value was comparable to that of the AJCC stage (Fig. 2e and Supplementary Fig. S2g). In addition, the data from TCGA dataset validated that lncSLCC1 expression increased in cancer tissues compared with corresponding normal tissues in CRC patients, and high lncSLCC1 expression predicted a poor prognosis (Fig. 2f, g). Besides, the clinicopathological analysis in TCGA dataset revealed that lncSLCC1 expression was positively correlated with lymph node invasion, metastasis, and AJCC stage (Fig. 2h). Collectively, lncSLCC1 is an lncRNA and highly expressed in CRC tissues.

### LncSLCC1 drives glycolytic metabolism to activate CRC proliferation

**Fig. 3:**
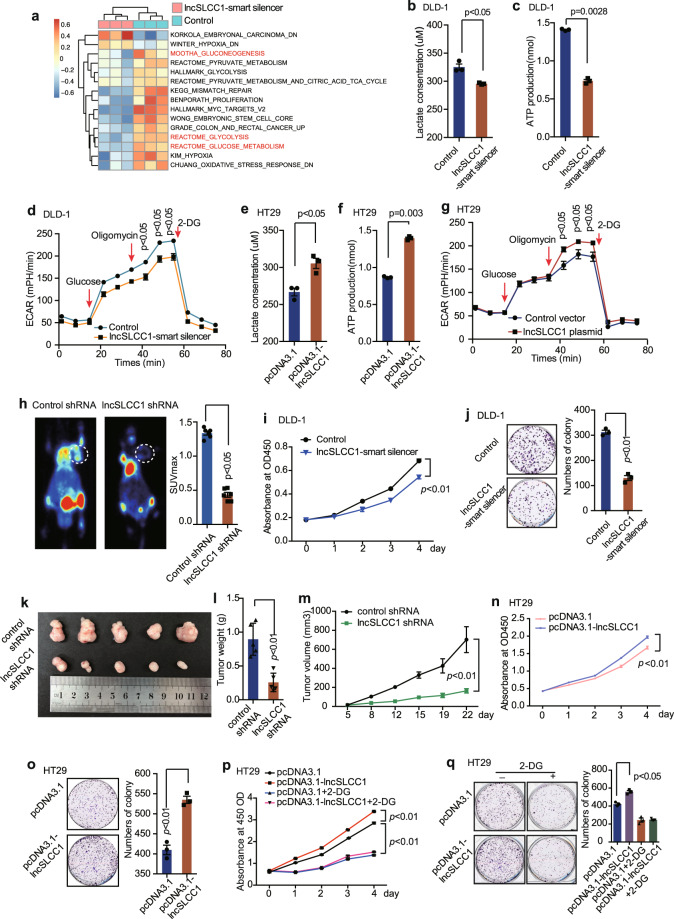
LncSLCC1 drives glycolytic metabolism to activate CRC proliferation. **a** ssGSEA analysis was performed to show the pathways closely correlated with lncSLCC1 expression in control and lncSLCC1 downregulated cells. **b**–**d** Lactate concentration (**b**), ATP production (**c**), and ECAR (**d**) were detected in control and lncSLCC1-knockdown DLD-1 cells. **e**–**g** Lactate concentration (**e**), ATP production (**f**), and ECAR (**g**) were measured in control and lncSLCC1-overexpression HT29 cells. **h** Representative images of 18F-FDG uptake by micro-PET imaging in control and lncSLCC1-knockdown xenograft mouse models. White circles indicated tumor glucose uptake. Maximum uptake values (SUVmax) for xenografts measured by FDG PET were presented. **i**, **j** Cell proliferation (**i**) and colony formation (**j**) were measured in DLD-1 cells after transfected with Control or lncSLCC1-smart silencer. **k**–**m** Representative images of tumors (**k**), tumor weights (**l**), and tumor volumes (**m**) were measured in nude mice bearing DLD-1 cells transfected with control shRNA or lncSLCC1 shRNA, *n* = 5. **n**, **o** Cell proliferation (**n**) and colony formation (**o**) were measured in HT29 cells after transfection with control or lncSLCC1-overexpression plasmid. **p**, **q** Cell proliferation (**p**) and colony formation (**q**) were measured in HT29 cells transfected with pCDNA3.1 and pCDNA3.1-*lncSLCC1* plasmid treated with or without glycolysis inhibitor 2-DG

To elucidate the potential mechanism lncSLCC1 involved in CRC tumorigenesis, we performed RNA-seq analysis to compare the gene expression profiles of lncSLCC1 knockdown and control DLD-1 cells (Supplementary Fig. S3a, b). A total of 267 downregulated genes and 66 upregulated genes (filter: *p* value < 0.005, fold change < 0.8 or > 1.2, base mean > 1000) were detected (raw data accessible via GEO number: GSE149783) after knockdown of lncSLCC1 in DLD-1 cells (Supplementary Fig. S3a and Supplementary Table S2). Gene set enrichment analysis (GSEA) of RNA-seq data showed that the gene signatures of glycolysis, colon and rectal cancer, and stem cell were enriched in DLD-1 control cells (Supplementary Fig. S3c–e). Single-sample GSEA (ssGSEA)^23^ further revealed that the gene sets related to glycolysis pathways and colorectal-carcinogenesis signatures were negatively correlated with lncSLCC1-knockdown CRC cells (Fig. 3a). Besides, the mass spectrometric metabolomics analysis was performed after knockdown of lncSLCC1 in DLD-1 cells. The high throughout metabolic analysis showed that knockdown of lncSLCC1 decreased the level of key components of glucose metabolism signaling, such as lactate (Supplementary Fig. S3f). These data indicate that lncSLCC1 may mediate glycolytic metabolism and carcinogenesis in CRC patients.

Further functional validation showed that lactic acid production (a key metabolite of glycolysis) and ATP production were both significantly decreased after lncSLCC1 knockdown in DLD-1 (Fig. 3b, c). Besides, we determined whether altered lncSLCC1 level directly influence glycolytic metabolism in CRC cells by measuring extracellular acidification rate (ECAR) and oxygen consumption rate (OCR). The knockdown of lncSLCC1 significantly reduced ECAR and OCR levels in DLD-1, compared with control cells (Fig. 3d and Supplementary Fig. S3g). In gain-of-function assays, overexpression of lncSLCC1 dramatically increased lactic acid production (Fig. 3e and Supplementary Fig. S3h), ATP production (Fig. 3f), ECAR (Fig. 3g), and OCR (Supplementary Fig. S3i) levels in HT29 cells. To further examine the effects of lncSLCC1 on glycolysis, we used 18F-FDG PET ([18F]-fluoro-2-deoxyglucose positron emission tomography) to measure glucose uptake in vivo. Mouse PET-CT data showed that knockdown of lncSLCC1 significantly reduced glucose uptake in the xenograft mouse tumor model (Fig. 3h and Supplementary Fig. S3j). These data indicate that lncSLCC1 may mediate glycolytic metabolism and carcinogenesis in CRC patients.

The previous GSEA of RNA-seq data showed that the gene signatures of colorectal carcinogenesis and stemness were enriched in DLD-1 cells transfected with Control compared to lncSLCC1-smart silencer (Supplementary Fig. S3c–e). The in vitro functional assays validated that knockdown of lncSLCC1 significantly impaired CRC cell proliferation and colony formation in DLD-1 cells (Fig. 3i, j) and HCT116 cells (Supplementary Fig. S3k, l). Besides, knockdown of lncSLCC1 dramatically reduced DLD-1 tumor growth (Fig. 3k), tumor weight (Fig. 3l), and tumor volume (Fig. 3m) in xenograft mouse tumor models in vivo. In the gain-of-function assays, overexpression of lncSLCC1 accelerated cell proliferation (Fig. 3n) and colony formation (Fig. 3o) in HT29 cells in vitro. In addition, knockdown of lncSLCC1 impaired CRC sphere formation in HCT116 and DLD-1 cells, which in accordance with the GSEA results (Supplementary Fig. S3m, n). Moreover, 2-DG (an inhibitor of glycolysis pathway) treatment significantly blocked lncSLCC1-induced cell proliferation (Fig. 3p) and colony formation (Fig. 3q). These data highly suggest that lncSLCC1 may promote CRC progression by activating glycolysis signaling.

### LncSLCC1 interacts with AHR and regulates HK2 expression

**Fig. 4:**
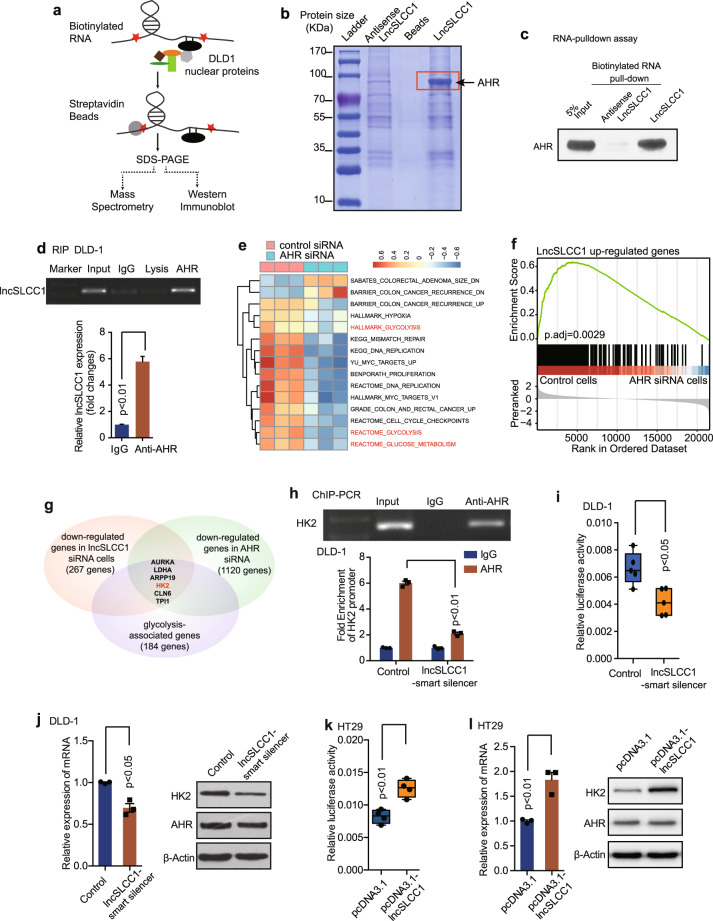
LncSLCC1 interacts with AHR and regulates HK2 expression. **a**, **b** Western blot of the proteins from antisense lncSLCC1 and lncSLCC1 pull-down assays. **c** Western blot of AHR in DLD-1 cells pulldown by lncSLCC1. **d** RNA immunoprecipitation experiments were performed using anti-AHR antibody, and specific primer was used to detect lncSLCC1. **e** ssGSEA analysis was performed to show the pathways closely correlated with AHR expression in control and AHR downregulated cells. **f** GSEA of lncSLCC1 upregulated genes in AHR-knockdown samples. Genes were ranked based on expression fold change between siCtrl and siAHR samples. **g** Venn diagram shows the genes downregulated after knockdown of lncSLCC1 (*p* < 0.005, fold change < 0.8, base mean > 1000, 267 genes), as well as AHR (*p* < 0.005, fold change < 0.8, base mean > 1000, 1120 genes), and associated with glycolysis (184 genes). **h** Real-time PCR of the ChIP samples shows the binding efficiency of AHR to the HK2 gene promoter in DLD-1 cells transfected with Control or lncSLCC1-smart silencer. **i** Luciferase reporter vectors were generated by inserting the promoter region (–2000 to 0 bp) of the HK2 gene. The reporter vectors were then co-transfected into DLD-1 cells with Control or lncSLCC1-smart silencer. Cells were harvested for luciferase activity assay. **j** The mRNA and protein levels of HK2 were detected in DLD-1 cells transfected with Control or lncSLCC1-smart silencer. **k** The luciferase activity was measured in HT29 cells co-transfected with HK2 promoter and control plasmid or lncSLCC1 plasmid. **l** The mRNA and protein levels of HK2 were detected in HT29 cells transfected with control plasmid or lncSLCC1 plasmid

Previously, we verified that lncSLCC1 was highly enriched in the nuclear fraction (Supplementary Fig. S2e). Nuclear lncRNAs can regulate target gene expression through a variety of mechanisms, commonly through interaction with protein complexes.^24^ Therefore, the identification of interacting proteins of lncSLCC1 is crucial for exploring its regulatory mechanism. For this purpose, we applied the pull-down technique in combination with mass spectrometry to screen the lncRNA interacting proteins. Biotinylated lncSLCC1 or antisense lncSLCC1 RNA (negative control) was incubated with total protein extracts from DLD-1 cells and pulled down with streptavidin (Fig. 4a). The associated proteins were analyzed by SDS-PAGE and Coomassie blue staining. There was a specific band in the lncSLCC1 pull-down sample (Fig. 4b). Mass spectrometry analysis identified the band as AHR (Supplementary Table S3), a transcriptional factor dysregulated in various types of cancer.^25^ We then validated this interaction between AHR and lncSLCC1 by western blotting following RNA pull-down (Fig. 4c). To verify the result, the anti-AHR antibody was used to immunoprecipitate endogenous AHR from cell lysate of DLD-1 cells and RNAs bound to AHR were extracted and analyzed. PCR data revealed that lncSLCC1 directly bound with AHR in CRC cells (Fig. 4d, upper panel). We also detected ~5-fold enrichments of lncSLCC1 in the anti-AHR immunoprecipitation, compared with the IgG control (Fig. 4d, down panel). The data suggest that AHR may interact directly with lncSLCC1.

To explore the function of AHR in CRC progression, we monitored cell proliferation after knockdown of AHR in the CRC cell line (Supplementary Fig. S4a, b). As with lncSLCC1, decreased AHR expression also led to a significant reduction in cell proliferation (Supplementary Fig. S4b). Mechanistically, GSEA and ssGSEA of RNA-seq data showed that knockdown of AHR also resulted in the downregulation of glycolysis associated pathways (Fig. 4e and Supplementary Fig. S4c).

To further investigate the function of the interaction between AHR and lncSLCC1, we tested the regulatory effect of AHR on the expression of lncSLCC1 target genes in DLD-1 cells. We performed RNA-seq in control and AHR-knockdown cells (raw data accessible via GEO number: GSE149867, Supplementary Table S4). The GSEA data demonstrate significant enrichment for the 267 lncSLCC1 upregulated genes in the control group (Fig. 4f). These data support our hypothesis on the convergence of AHR and lncSLCC1 pathways in upregulating downstream glycolysis genes which ultimately promote CRC progression. Further analysis showed HK2, which was the genes involved in the glycolysis pathway, was simultaneously downregulated in AHR-knockdown as well as lncSLCC1-knockdown cells (Fig. 4g and Supplementary Fig. S4d, e). It has been reported that high levels of HK2 correlated with poor clinical outcome in CRC, and this gene may regulate glycolytic metabolism, and then promote cancer cell proliferation and invasion.^26^ This suggests that lncSLCC1 may bind with AHR and actively regulate HK2 gene expression, and ultimately regulate glycolysis and cell proliferation. To confirm the assumption, we found that there were two predicted binding sites of AHR on the promoter sequence of target gene HK2 (http://alggen.lsi.upc.es/home.html) (Supplementary Fig. S4f). ChIP-PCR further indicated that AHR might bind to the promoter of HK2 (Fig. 4h), and the binding efficiency of AHR on HK2 promoter was significantly decreased in lncSLCC1 downregulated DLD-1 cells (Fig. 4h). We next examined the effect of lncSLCC1 on the transcriptional activity of HK2 gene. Luciferase assay showed that knockdown of lncSLCC1 decreased the transcriptional level of HK2 promoter in DLD-1 (Fig. 4i). Real-time PCR and western blot data revealed that HK2 expression was significantly downregulated in DLD-1 (Fig. 4j) and HCT116 (Supplementary Fig. S4g) cells after transfection with lncSLCC1-smart silencer. In accordance with the data, downregulation of AHR expression level also decreased the transcriptional level and expression of HK2 (Supplementary Fig. S4h, i). In addition, in the gain-of-function assays, overexpression of lncSLCC1 promoted the transcriptional level of HK2 and increased HK2 expression in HT29 cells (Fig. 4k, l). These data indicate that lncSLCC1 may positively regulate HK2 transcription in CRC cells.

### HK2 is the functional target gene of lncSLCC1 in CRC

**Fig. 5:**
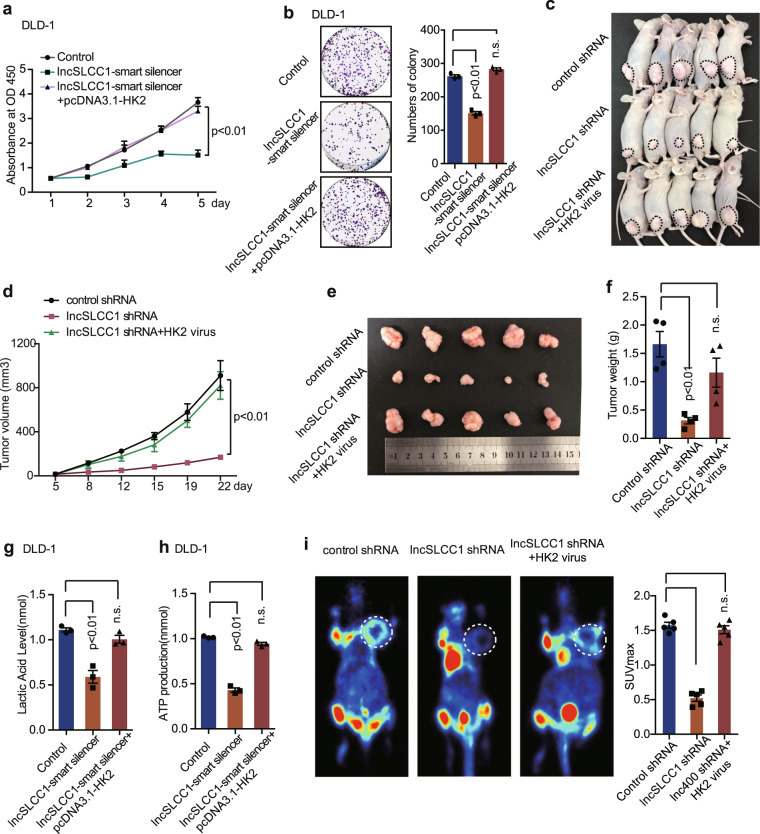
HK2 is the functional target gene of lncSLCC1 in colorectal cancer. **a**, **b** CCK8 assays (**a**) and colony formation assay (**b**) were measured after transfection with Control, lncSLCC1-smart silencer, and lncSLCC1-smart silencer with HK2 plasmid in DLD-1 cells. **c**–**f** Representative images of tumors (**c**), statistical analysis of tumor volumes (**d**, **e**) and tumor weights (**f**) in nude mice bearing DLD-1 cells in different groups (*n* = 5, nonparametric Mann–Whitney test). **g**, **h** The lactic acid level and ATP production were measured in DLD-1 cells transfected with Control, lncSLCC1-smart silencer, and lncSLCC1-smart silencer with HK2 plasmid. **i** Representative images and summarized data of ^18^F-FDG uptake by micro-PET imaging in control shRNA, lncSLCC1 shRNA, and lncSLCC1 shRNA with HK2 virus in xenograft mouse models (inoculated in the armpit of mice, pictures were taken in the layer which showed similar FDG uptake in non-tumor regions among various group). White circles indicated tumor glucose uptake. Maximum uptake values (SUV_max_) for xenografts measured by FDG PET were presented, nonparametric Mann–Whitney test

Hexokinase 2 (HK2) is a pivotal kinase in the glycolytic pathway.^27^ Previous studies have demonstrated that HK2 activity is remarkably increased in various malignant neoplasms, as well as in CRC.^28^ Our previous data showed that lncSLCC1 could activate glycolysis, promote CRC, and regulate HK2 expression. We next examined whether HK2 mediated the biological function of lncSLCC1 in CRC. First, DLD-1 cells were transfected with control, lncSLCC1-smart silencer or co-transfected with lncSLCC1-smart silencer and HK2-overexpression plasmids. The in vitro functional assay demonstrated that overexpression of HK2 significantly rescued lncSLCC1 downregulation induced decrease in cell proliferation (Fig. 5a) and colony formation (Fig. 5b). Then, DLD-1 cells were injected into the mice subcutaneously to establish the CRC xenograft model. And the mice were injected with control, lncSLCC1 shRNA, or lncSLCC1 shRNA and HK2-overexpression virus by multipoint intratumoral injection. The in vivo data indicated that upregulation of HK2 significantly delivered lncSLCC1 downregulation induced decline in the tumor growth (Fig. 5c–e) and tumor weight (Fig. 5f). To verify the function of HK2 in lncSLCC1-mediated activation in glycolysis metabolism, the lactic acid level, and ATP production were detected in DLD-1 cells transfected with control, lncSLCC1-smart silencer or co-transfected with lncSLCC1-smart silencer and HK2-overexpression plasmids. The results demonstrated that overexpression of HK2 rescued lncSLCC1 downregulation led decreased in lactic acid level (Fig. 5g) and ATP production (Fig. 5h) in vitro. In addition, the in vivo assay further verified that overexpression of HK2 significantly rescued lncSLCC1 downregulation induced decrease in glucose uptake (Fig. 5i and Supplementary Fig. S5a, b). Our data thus indicate that lncSLCC1-regulated glycolytic metabolism and cell proliferation mediated by HK2 in CRC.

### LncSLCC1 and HK2 expression correlates and clinically relevant in CRC patients

**Fig. 6:**
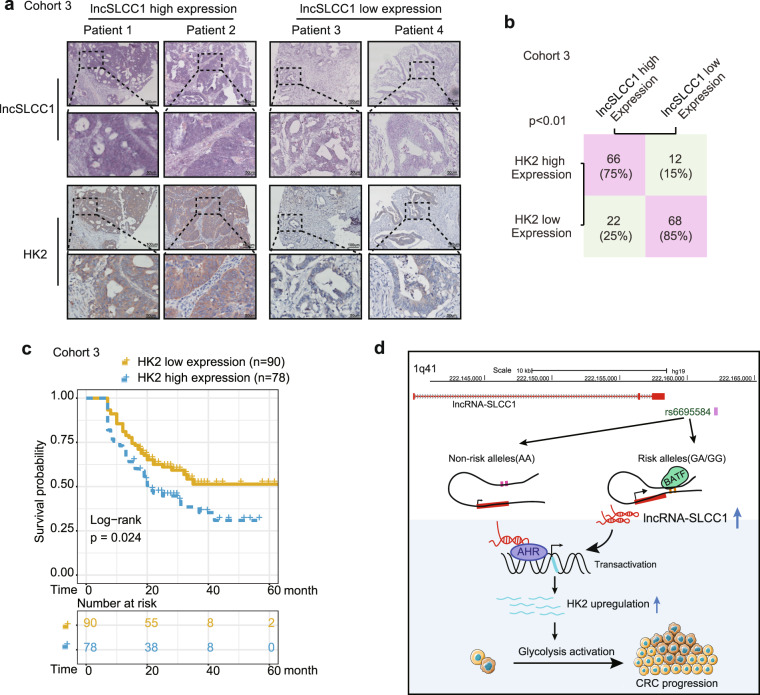
LncSLCC1 and HK2 expression correlates and clinically relevant in CRC patients. **a** The representative images of ISH of lncSLCC1 and immunohistochemical staining of HK2 in Cohort 3. **b** Correlation between lncRNA-SLCC1 and HK2 expression in CRC tissues of Cohort 3. **c** Kaplan–Meier curves showing the overall survival of CRC patients with different levels of HK2 protein intensity in Cohort 3. **d** Schematic representation for the mechanism of SNP/lncSLCC1/AHR/HK2 axis as a switch that regulates glucose metabolism in human colorectal cancer progression

To explore the clinical correlation between lncSLCC1 and HK2. We performed immunohistochemical staining in CRC patients’ tissues of Cohort 3. Expectedly, the samples with lncSLCC1 higher expression displayed strongly staining for HK2 (Fig. 6a, left panel). In addition, samples with low expression of lncSLCC1 appeared low levels of HK2 (Fig. 6a, right panel). The data are statistically significant (Fig. 6b). The data indicate that lncSLCC1 expression is positively correlated with HK2 expression in CRC tissues. We next assessed the association between the intensity of HK2 and overall survival rate in these CRC patients of Cohort 3. This analysis showed that elevated expression of HK2 in CRC tissues predicted robustly shorter survival time (Fig. 6c). These data demonstrate that elevated expression levels of lncSLCC1 and its target gene HK2 may identify CRC patients with poor prognosis.

Taken together, our data suggest a model that rs6695584 risk allele elevates the abundance of lncSLCC1, and then leads to increased formation of AHR-lncSLCC1 complex, resulting in subsequent upregulation of target gene HK2, ultimately promotes CRC development and progression.

## Discussion

GWAS have identified thousands of SNPs associated with diseases, most of which are located in non-coding regions.^29^ In the post-GWAS era, revealing the molecular basis and biological function of the trait-associated SNPs has become a great challenge. Although more than 50 SNPs have been demonstrated to contribute to the development and progression of CRC, the underlying mechanism remains unclear.^8^ In the previous study, tag SNP rs6687758 was found to be a risk loci for CRC.^11^ However, the causative SNP and the function of this region were inclusive. Here, we revealed the functional mechanisms and clinical implications of the causative SNP rs6695584 in the progression of CRC, which promoted the expression of lncSLCC1 as an enhancer element by targeting a transcriptional factor BATF. As a consequence, upregulated lncSLCC1 activated the glucose metabolism reprogramming through the AHR–HK2 pathway.

The clinical stage and tumor grade are the most common risk factors for the prognosis of patients with CRC. With the rising of molecular classification of cancer, several nomograms for survival and response to therapy in CRC had taken the gene signatures into the formula.^30^ In our results, the G allele of rs6695584 was associated with poorer overall survival than the A allele, suggesting that the genotype of this risk loci might serve as a predictor of the clinical outcome of CRC patients. Analogously, Fu et al.^31^ demonstrated that CRC patients with genotypes TT/CT of rs12982687 enjoyed a more favorable prognosis than those with homozygote C. Genetic background combined with molecular biomarkers and clinical features could construct a more accurate model for the outcome of CRC patients.

The function of disease-associated SNPs differs in their location on the genome. Several SNPs located in protein-coding regions could influence the biological process through directly regulating protein expression,^10,32^ while those in non-coding regions had a more comprehensive regulatory mechanism. Gupta et al.^33^ illustrated that some non-coding variants within potential regulatory elements could distally regulate target gene expression and contribute to the pathogenesis of multiple diseases. The regulatory elements could be either promoters or enhancers depending on their locations, associated histone marks and regulatory effects, or even a bifunctional regulatory element that can function as both a promoter and an enhancer.^17^ In our study, dual-luciferase-based enhancer assays and ChIP-seq data suggested that the risk allele of rs6695584 modified the enhancer of lncRNA-SLCC1 and upregulated its expression. Motif analysis showed that rs6695584 changed the binding motif of BATF, which is an oncogenic TF described in types of cancers.^34,35^ BATF is one of the activator protein 1 (AP-1) TFs and the AP-1-BATF module could upregulate several marker genes such as IL17F, IL22, IL26, and IL23R in anaplastic large cell lymphoma.^35^ Here, the variation of risk SNP rs6695584 increased the binding of BATF with enhancer element, and consequently promotes the expression of lncSLCC1. The results showed that the rs6695584 enrichment in GG (RKO) is not higher than that in AG (DLD-1) in Fig. 1i, which may due to the heterozygous genotype.^36,37^ In line with our results, Gao et al.^38^ found that SNP rs11672691 also functioned as an enhancer for CEACAM21 in promoting prostate cancer aggressiveness. Thus, modification of regulatory elements might be a common mechanism of gene expression referring to intergenic SNPs.

With the development of whole genome and transcriptome sequencing in the last 20 years, our understanding of lncRNAs in the pathogenesis of cancer has improved greatly. Mechanistically, lncRNAs can interact with biological molecules, like DNA domain, RNA, and proteins, and regulate chromatin conformation, transcriptional, and post-transcriptional expression of target genes.^39^ In this study, we found that the expression of lncSLCC1 was significantly increased in cancer tissues than adjacent normal tissues of CRC patients and was a predictive biomarker for poor prognosis, which was validated in two independent cohorts. In supportive of our data, previous studies also confirmed that lncSLCC1 (RP11-400N13.2) was positively correlated with overall survival^40,41^ and was explanatory lncRNA for TASs for CRC.^42^ Furthermore, the in vivo and in vitro experiments demonstrated that lncSLCC1 could regulate glucose metabolism reprogramming and promote carcinogenesis in CRC patients.

Dysregulation of glucose metabolism has been proven as a hallmark of cancer cells.^43^ Numerous studies have demonstrated that lncRNAs can participate in glucose metabolism by regulating signaling pathways (like HIF signaling pathway, PI3K/AKT/mTOR signaling pathway, and Wnt/snail signaling pathway) or regulating crucial molecules, key enzymes, and oncogenes involved in glucose metabolism.^44^ In our case, we revealed that lncSLCC1 could bind to AHR and actively regulate HK2 gene expression, which is a key enzyme in the process of glycolysis, consequently, activating glycolysis metabolism and promote CRC progression. All the above studies suggested that lncRNAs and lncRNA-mediated metabolic pathways are a crucial part of CRC development. A fully understanding of their role in CRC may provide novel targets for cancer therapy.

In conclusion, we elucidated the functional mechanism of CRC-associated risk SNP rs6695584 in CRC progression (Fig. 6d). Mechanistically, we revealed a risk SNP-modulated enhancer alteration in upregulating lncRNA-SLCC1. Functionally, lncSLCC1 interacts with AHR to upregulate glycolysis-related gene HK2, ultimately contributing to CRC cell growth and progression. Given the genetic, clinical, and functional significance of 1q41 and the defined target gene, lncSLCC1, we conclude that the SNP rs6695584/lncSLCC1/HK2 pathway is crucial for colorectal progression, and targeting this pathway may be pivotal in the prevention or treatment of CRC.

## Materials and methods

### Patient specimens

We used one Cohort of health control patients who had undergone colonoscopy between 2015 and 2018 (Cohort 1, fresh tissues). We used two cohorts of CRC patients who underwent surgery between 2012 and 2018 (Cohorts 2 and 3). Cohort 2 (fresh tissues) were from the Eastern Campus of Renji Hospital, Shanghai Jiao Tong University School of Medicine; Cohort 3 (paraffin-embedded tissues) was from the Western Campus of Renji Hospital, Shanghai Jiao Tong University School of Medicine. All the patients were Han Chinese and none of these patients had received radiotherapy or chemotherapy prior to surgery. The study protocol was approved by the ethics committee of Renji Hospital, Shanghai Jiao Tong University School of Medicine. Written informed consent was obtained from all participants in this study. All the research was carried out in accordance with the provisions of the Declaration of Helsinki of 1975.

### Total RNA extraction and real-time PCR

Total RNA of CRC tissues and cell lines was extracted by trizol reagent (Takara, Japan). One microgram total RNA was reverse transcribed by PrimeScript RT Reagent Kit (Takara, Japan). Real-time PCR was performed using ABI reagent (Thermo Fisher Scientific, West Palm Beach, FL) by StepOne Plus real-time PCR system (Applied Biosystems, Foster City, CA). 2^−ΔΔCt^ method was used to quantify the relative expression. β-Actin was used as an internal control. All the primers sequences used in this study are listed in Supplementary Table S5.

### eQTL analysis

The relative expression of lncSLCC1 and genotypes of rs6695584-G/A were analyzed in adjacent normal colorectal mucosa. The eQTL analysis was performed by fitting a linear regression model between the expression levels of lncSLCC1 and the genotype of rs6695584 using the R package “MatrixEQTL” version 2.1.^45^ For the TCGA cohort, the level 3 RNA-seq data and the genotyping data of Affymetrix Genome-Wide Human SNP Array 6.0 were obtained from the TCGA data hub.

### In vitro transcription and translation

lncSLCC1, lncRNA GLCC1, and HSP90 were cloned into pBluescript KSII downstream of the T7 promoter. The plasmids were transcripted (Promega, Madison, WI) and purified (Qiagen, Germany) in vitro. Biotinylated leucine tRNA (Promega, Madison, WI) was used to translate the purified RNAs in vitro. BrightStar BioDetect Kit (Ambion, West Palm Beach, FL) was used to detect the biotinylated proteins. LncRNA GLCC1 and water were used as negative controls and HSP90 mRNA was used as a positive translation control.

### Plasmids and adenovirus construction

The control-overexpressing plasmid, lncSLCC1-overexpressing plasmids, and HK2-overexpression plasmids were constructed by Generay Technologies (Shanghai, China). The shControl lentivirus, sh-lncSLCC1 lentivirus, control lentivirus, and HK2 lentivirus were constructed by Obio Technology Company (Shanghai, China).

### In vivo xenograft model

Four-week-old male BALB/c nude mice were purchased from Experimental Animal Center of SIBS. DLD-1 cells were injected subcutaneously in the groin or armpit into the mice to establish the CRC xenograft model (for the tumor formation assay, cells were inoculated in the groin of mice; for the ^**1**8^F-FDG PET detection assay, which were repeated for two times, cells were inoculated in the groin or the armpit of mice, separately). Five days after inoculation, the mice were injected with indicated adenovirus by multipoint intratumoral injection every 2 days. The mice were sacrificed 3 weeks after the inoculation of tumor. Tumor volume (mm^3^) was assessed by the formula: tumor volume (mm^3^) = longer diameter × shorter diameter^2^/2.

### Lactate production and ATP production

l-Lactate Assay kit (Colorimetric) was used to measure the lactate production (Abcam, Cambridgeshire, UK) according to the manufacturer’s protocols. The transfected cells were planted into 96-well cell culture plates and incubated at 37 °C overnight. After starvation for 2 h, the supernatant was collected for measurement of lactate production. The lactate production levels were measured at 450 nm in a microplate reader.

ATP Assay kit (Colorimetric) was used to measure the ATP production (Abcam, Cambridgeshire, UK) according to the manufacturer’s protocols. CRC cells were seeded into six-well plates and transfected with indicated constructs and incubated for 48 h. 1 × 10^6^ transfected cells were harvested and homogenized with ATP assay buffer. After centrifuged for 5 min, the supernatant was collected for measurement of ATP production. The ATP production levels were measured at 570 nm in a microplate reader.

### ^18^F-FDG PET imaging

Four-week-old male BALB/c nude mice were subcutaneously injected with DLD-1 cells in the groin or the armpit and injected with indicated adenovirus. Three weeks after tumor implantation, the mice were used for ^18^F-FDG PET imaging. Briefly, mice were fasted for 8 h and injected with approximately 10 μCi ^18^F-FDG per gram of mouse via lateral tail vein. The exact dose of ^18^F-FDG was calculated by measuring the syringe before and after injection. Mice were maintained in cages at room temperature for 1 h and then anesthetized with isoflurane. Mice were subjected to micro-PET and micro-CT imaging on a warm pad in the prone position. ^18^F-FDG uptake was quantified by drawing region of interest using AMIDE software and plotting maximum uptake values (SUV_max_).

### RNA immunoprecipitation

RNA immunoprecipitation (RIP) assays were conducted using the Magna RIP Kit (Millipore, New Bedford, MA) according to the manufacturer’s protocols. DLD-1 cells were seeded into a 10-cm culture dish. Cells were prepared using RIP lysis buffer and the RNA–protein complexes were immunoprecipitated using anti-AHR antibody (CST, Boston, MA) and normal rabbit IgG. The co-precipitated RNAs were purified using phenol:chloroform:isoamyl alcohol and subjected to reverse transcription-PCR or real-time PCR analysis. A control amplification was carried out on the input RNA before immunoprecipitation.

### Chromosome conformation capture combined with quantitative PCR (3C-qPCR)

2 × 10^7^ cells (Caco2, DLD-1, RKO) were fixed with 1% formaldehyde and fractionated for nuclear fraction. The nuclear lysates were incubated with 400 U *Pst*l at 37 °C overnight for digestion. The digested chromatin was incubated with a ligation buffer at 16 °C overnight. The ligation buffer consisted of 750 μL of 10× NEB ligation buffer, 750 μL of 10% Triton X-100, 75 μL of 10 mg/ml BSA, 4000U T4 DNA ligase, and 5925 μL of distilled water (NEB, Hertfordshire, UK). DNA was extracted using phenol–chloroform. Extracted DNA was used for further PCR and qPCR amplification.

### Luciferase assay

The fragments were synthesized each into the cloning site of pGL3-LRF and pGL3-LRR at Generay Technologies (Shanghai, China). CRC cells were seeded in 96-well plates and transfected with 500 ng indicated plasmids and 100 ng pRL-TK plasmid (Renilla luciferase) using FuGene HD (Promega, Madison, WI). The relative firefly luciferase activity and Renilla luciferase activity were detected using the dual-luciferase reporter assay system (Promega, Madison, WI) and measured by FLUOstar Omega (BMG LABTECH, Offenburg, Germany) 24 h after transfection. The results were shown in the form of relative firefly luciferase activity normalized to Renilla luciferase activity. pGL3-control vector was used as a positive control and pGL3-LRF and pGL3-LRR were used as negative controls.

### Motif analysis

FIMO (http://meme-suite.org/tools/fimo) and JASPAR (http://jaspar.genereg.net/) were used to analyze the effect of rs6695584 on the TF-binding motifs. SNP rs6695584 and its flanking sequences overlapped with BATF motifs.

### RNA pull-down assay

RNA pull-down assay was performed using synthesized biotinylated lncSLCC1 or antisense lncSLCC1 as a probe. The diluted RNAs were incubated at 60 °C for 10 min, slowly cooled to 4 °C, and then used for pull-down experiments. DLD-1 cells were harvested into 5 mL of buffer A [10 mM Tris·HCl, pH 7.0, 1.5 mM MgCl_2_, 10 mM KCl, 0.5 mM DTT, 1 mM PMSF, and protease inhibitor mixture (Roche Molecular Biochemicals, Mannheim, Germany)]. In all, 0.25% Nonidet P-40 was used to lyse the cells. The lysates were centrifuged and the supernatant was removed. The samples were resuspended in 3 mL of buffer C (25 mM Tris·HCl, pH 7.0, 0.5% Nonidet P-40, 150 mM KCl, 0.5 mM DTT, and protease inhibitor mixture) and sheared by homogenizing for 15–20 strokes. The samples were centrifuged and the supernatant was removed. The nuclear lysates containing 1 mg of protein were precleared with streptavidin beads and then incubated with 2 μg of biotinylated RNA (lncSLCC1 or antisense lncSLCC1) and 40 μl of streptavidin beads for 2 h at 4 °C. Beads were collected by centrifugation and washed with buffer C for three times. RNA-bound proteins were eluted and resolved by SDS/PAGE followed by silver staining (Biorad, Hercules, CA).

### Chromatin immunoprecipitation

Chromatin immunoprecipitation (ChIP) assays were conducted using the ChIP Assay Kit (Millipore, New Bedford, MA) according to the manufacturer’s protocols. DLD-1 cells were seeded into a 10 cm culture dish. 2 × 10^7^ cells were fixed with 1% formaldehyde and quenched with 0.125 M glycine. Cells were then collected using SDS lysis buffer and sonicated to shear DNA to lengths between 200 and 800 base pairs. The DNA–protein complexes were precleared with Protein A Agarose/Salmon DNA and then immunoprecipitated with anti-AHR antibody (CST, Boston, MA), anti-Pol II antibody (CST, Boston, MA), and normal rabbit IgG. The co-precipitated DNAs were purified using phenol/chloroform. The extracted DNA was used for further PCR and qPCR analysis.

### High-throughput sequencing

For RNA sequencing, samples were run on an Illumina HiSeq 4000 for 2 × 150-bp paired-end sequencing. The RNA-seq data analysis was performed according to the Hisat- Featurecounts-DeSeq2 frame. Briefly, the fastq files were then map-ped to the human hg19 reference genome using HISAT2 (version 2.1.0)^46^ and read counts were obtained using subread featureCounts (version 1.6.3).^47^ The read counts from each sequenced sample were combined into a count file, which was subsequently used for the differential expression analysis. The read counts from each sequenced sample were combined into a count file, which was subsequently used for the differential expression analysis. Differential analyses were performed to the count files using DESeq2 packages, following standard normalization procedures.^48^ Genes with less than five total counts in both conditions were removed from further analysis.

### Statistical analysis

Statistical analyses were carried out using the program R (www.r-project.org). Data were presented as the mean ± SEM. Data were examined to determine whether they were normally distributed with the one-sample Kolmogorov–Smirnov test. If the data were normally distributed, comparisons of measurement data between two groups were performed using independent sample *t-*test and the comparisons among three or more groups were performed by one-way ANOVA test. If the results showed a significant difference, when the data were skewed distribution, comparisons were performed by nonparametric test. Measurement data between two groups were performed using the nonparametric Mann–Whitney test. Overall survival was evaluated by the Kaplan–Meier survival curve and log-rank tests. Correlation between lncRNA-SLCC1 expression and HK2 expression in CRC patients was examined by chi-square test. ssGSEA was calculated by Gene Set Variation Analysis (GSVA) R package. *P* < 0.05 was considered to be statistically significant.

## Supplementary information

Supplementary materials

Supplementary Table S1

Supplementary Table S2

Supplementary Table S3

Supplementary Table S4

Supplementary Table S5

## Data Availability

The datasets used during the current study are available at the Gene Expression Omnibus (GSE149783, GSE149867). The ChIP-Seq data used in Fig. 1h can be accessed at the ENCODE database. Other methods are available in the additional Supplementary Methods file.
