# Supplementary material for: Risk SNP-induced lncRNA-SLCC1 drives colorectal cancer through activating glycolysis signaling
Source: Signal Transduct Target Ther. 2021 Feb 19;6:70. doi: 10.1038/s41392-020-00446-7 (PMC7892549; doi:10.1038/s41392-020-00446-7)
Supplement: Supplementary file 1 — Supplementary materials [file 41392_2020_446_MOESM1_ESM.docx]

Supplementary Materials for

**Risk SNP-induced lncRNA-SLCC1 drives colorectal cancer through activating glycolysis signaling**

Tingting Yan^1†^, Chaoqin Shen^1†^, Penglei Jiang^2†^, Chenyang Yu^1^, Fangfang Guo^1^, Xianglong Tian^1^, Xiaoqiang Zhu^1^, Shiyuan Lu^1^, Bingshe Han^2^, Ming Zhong^3^, Jinxian Chen^3^, Qiang Liu^4^, Yingxuan Chen^1^, Junfang Zhang^2^*, Jie Hong^1^*, Haoyan Chen^1^*, Jing-Yuan Fang^1^*

Correspondence to J.F: jingyuanfang@sjtu.edu.cn; H.C: haoyanchen@sjtu.edu.cn; J.H: jiehong97@sjtu.edu.cn; J.Z: jfzhang@shou.edu.cn

**This PDF file includes:**

Supplementary Methods

Supplementary Figures. S1 to S5

**Other Supplementary Materials for this manuscript include the following:**

Supplementary Tables S1 to S4

**Supplementary Methods**

**Immunohistochemistry and in situ hybridization**

For the immunohistochemistry assay, CRC tissue microarray sections were rehydrated and treated with hydrogen peroxide. Heat-mediated antigen retrieval was carried out by microwave with sodium citrate. The slides were incubated with primary antibody against HK2 (CST, Boston, MA, 1:200 dilution) on a humidified box at 4℃ overnight. DAB substrate kit was used for the color-reaction, and hematoxylin was used for nucleus counterstaining.

In situ hybridization detection of lncSLCC1 was performed on 6-μm formalin-fixed, paraffin-embedded (FFPE) sections using DIG-labeled miRCURYTM Detection probe (Exiqon, Denmark). Briefly, the slides were hybridized with a probe (LNA-modified and DIG-labeled oligonucleotide; Exiqon, Denmark) complementary to lncSLCC1 and after incubation with anti–DIG-AP Fab fragments conjugated to alkaline phosphatase. Nitroblue tetrazolium/5-bromo-4-chloro-3-indolyl phosphate color substrate (Roche, Switzerland) was used for color detection of the hybridized probes. Finally, the slides were counterstained with VECTOR nuclear fast red counterstain (Vector Laboratories, China).

The expression of HK2 protein and lncSLCC1 mRNA was assessed according to the intensity and extent of staining at 200X under microscope. The intensity of staining score was evaluated on a scale of 0-3: 0=no staining; 1=weak staining; 2=moderate staining; 3=strong staining. The extent score presenting the percentage of positively stained cells (0=0-5%; 1=6-25%; 2=26-50%; 3=51-75%; 4=76-100%). The final score (protein expression) was represented as the multiplication product of intensity score and extent score. The tissues with a final score ≤6 were classified as “Low Expression” and the tissues with a final score >6 were classified as “High Expression”.

**Western blot**

Protein was extracted by RIPA lysis buffer containing a protease inhibitor mixture (protease inhibitors; phosphatase inhibitors; PMSF; KangChen, China). The concentration of protein was quantified by BCA Protein Assay Kit (Thermo Fisher Scientific, West Palm Beach, FL). 40 μg of protein was separated by 10% SDS-polyacrylamide gels and then transferred to PVDF membranes (Biorad, Hercules, CA). After blocked with 5% BSA for 2 hours, the membranes were incubated with primary antibody rabbit anti HK2 (1:1000 dilution, CST, Boston, MA), anti AHR (1:1000 dilution, CST, Boston, MA), anti β-actin (1:20000 dilution, Sigma, Louis, MO) at 4 ℃ overnight. Then, the membranes were washed with TBST for 5 times and incubated with species-specific secondary antibodies (1:3000 dilution, Kangcheng, China) for 1 hour the next day. Secondary antibodies were labeled with HRP. The ECL detection system (Biorad, Hercules, CA) was used for visualization. Antibody against β-actin was used as an internal control.

**Cell proliferation, colony formation and sphere formation assay**

For the cell proliferation assay, control and transfected CRC cells were planted into 96-well culture plates (3000 cells/well). Cell Counting Kit-8 (Dojindo, Japan) was added to the cells at specific time points (24h, 48h, 72h, 96h and 120h after cell plantation). After incubated with CCK-8 reagent for 2 hours away from light, the absorbance was measured by OD at 450 nm wavelength. For the colony formation assay, control and transfected CRC cells were seeded into 6-well culture plates (800 cells/well). After 7 to 10 days of incubation, the cells were fixed with 4% paraformaldehyde for 20 minutes, stained with 0.1% crystal violet for 20 minutes, washed with PBS for 5 times and air-dried. Finally, the colonies were counted. For the sphere formation assay, control and transfected CRC cells were planted into 96-well ultra-low-attachment culture plates (800 cells/well). The cells were cultured in DMEM/F12 medium supplemented with B27 supplement, 20ng/ml EGF (epithelial growth factor), 20ng/ml bFGF (fibroblast growth factor) and insulin. The number of spheres was counted after 10 days.

**Mass spectrometric metabolomics analysis**

DLD1 cells were seeded into 10cm culture dishes and transfected with indicated constructs. 48 hours after transfection, the cells (2×10^7^ cells）were collected and added with 1mL pre-cooled methanol/acetonitrile/water (2:2:1, v/v/v). The samples were vortexed, sonicated for 20 minutes at 4 °C and incubated for 1 hour at -20 °C to precipitate proteins. The sample was then centrifuged at 14000g for 15 minutes at 4 ℃ and the protein was dried under vacuum. For mass spectrometry, the dried samples were dissolved in 100 μL of acetonitrile-water solution (1:1, v/v) and centrifuged at 14,000g for 15 min at 4 °C. Electrospray ionization was conducted using Agilent 1290 Infinity chromatography system and QTRAP 5500 (AB SCIEX) mass spectrometer. A QC (quality control) sample is set every 3 experimental samples in the experiment, which is used to evaluate the stability and repeatability of the system. Multiquant software was used to measure chromatographic peak area and retention time. Standards for energy metabolites are used to correct and normalize the retention time, and then identify the metabolite.

**Seahorse analysis**

CRC cells were seeded into the 96-well cell culture plates and then the cells were used for measurement of ECAR and OCR. Seahorse XF Glycolysis Stress Test Kit was used to measure ECAR levels. Briefly, glucose, oligomycin, and 2-DG were sequentially added into each well for ECAR measurement after measurement of baseline concentration. Seahorse XF Cell Mito Stress Test Kit (Agilent Technologies, Palo Alto, CA) was used to measure OCR levels. Oligomycin, FCCP (p-trifluoromethoxy carbonyl cyanide phenylhydrazone), and Antimycin A & Rotenone were sequentially injected into each well for the measurement of OCR levels. Seahorse XF-96 Wave software was used to analyze the data.

**Supplementary Figures**

**
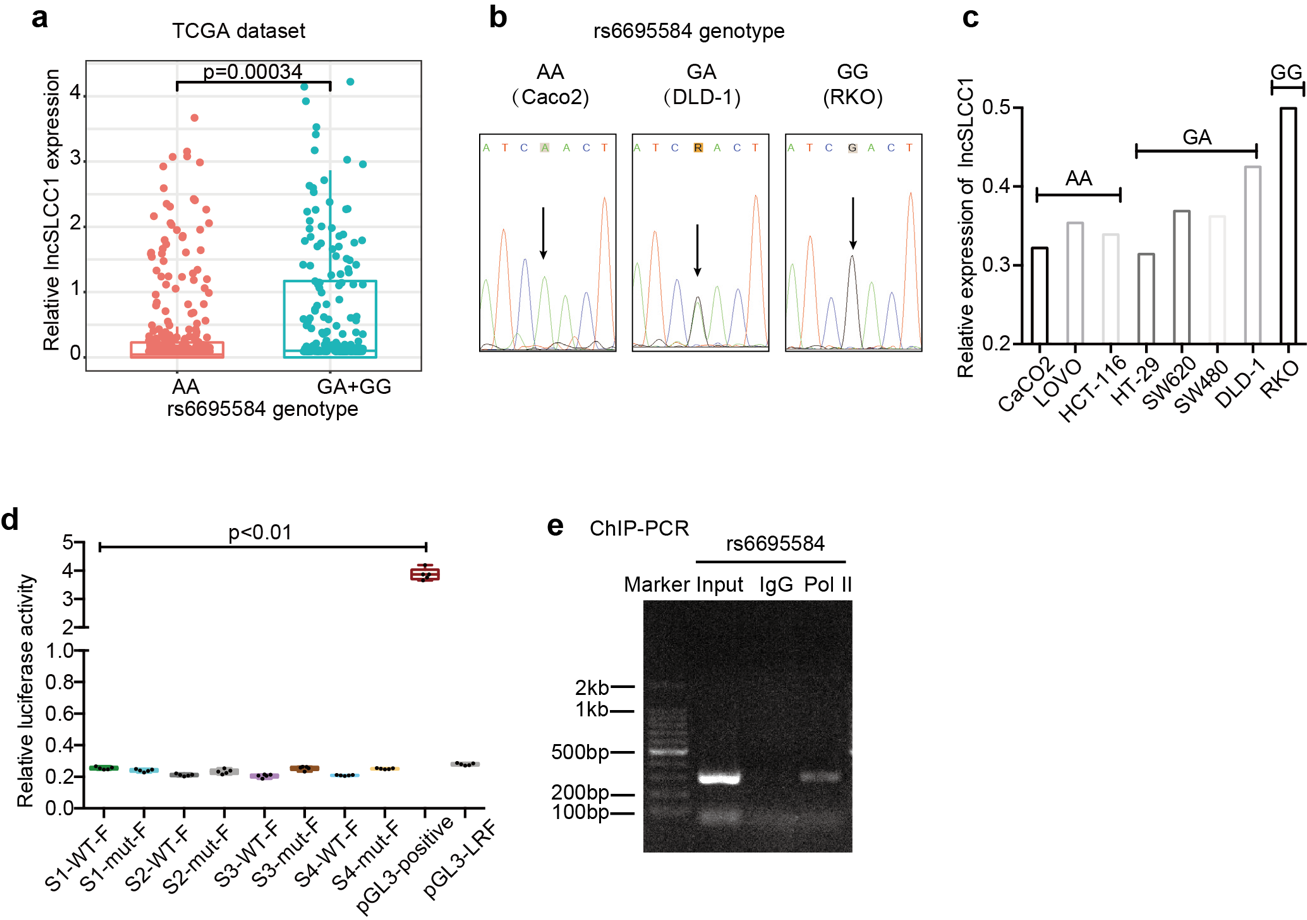
**

**Supplementary Fig. S1. CRC associated risk SNP rs6695584 regulates lncSLCC1 expression as an enhancer. (a)** The expression of lncSLCC1 in normal colon mucosa with different genotypes of SNP rs6695584 in TCGA dataset. **(b)** Sanger sequencing of the SNP rs6695584 in Caco2 (AA), DLD-1 (GA) and RKO (GG) cells. **(c)** The expression of lncSLCC1 in CRC cells with different genotypes of SNP rs6695584. **(d)** Relative reporter gene activity of the constructs containing the WT or Mut allele of different SNPs in forward orientation in DLD-1 cells. **(e)** Polymerase II ChIP-PCR gel of rs6695584 in DLD-1cells.

**
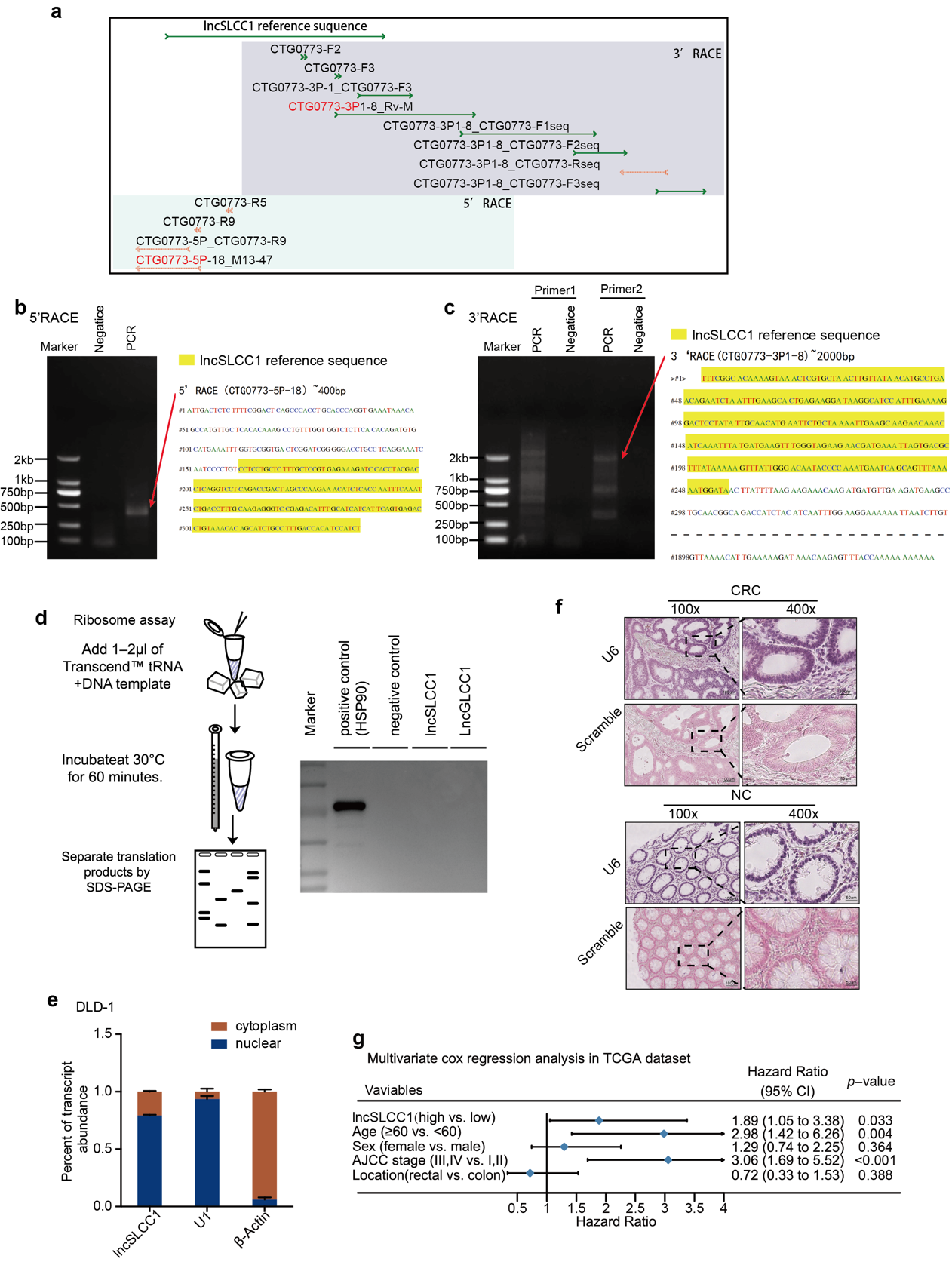
**

**Supplementary Fig. S2. LncRNA-SLCC1 is clinically relevant in colorectal cancer. (a)** Representative images of 3’-Race and 5’-Race PCR products of lncSLCC1 are shown. **(b)** The red line shows the sequence between the universal anchor primer and lncSLCC1. **(c)** The ribosome translation assay was performed in positive control HSP90, negative control, lncSLCC1 and lncGLCC1. **(d)** Expression of lncSLCC1 in cytoplasmic and nuclear fractionations of colorectal cancer cells. U1 RNA serves as a positive control for nuclear gene expression and β-actin serves as a positive control for cytoplasmic gene expression. **(e)** The representative ISH images of U6 (positive control) and Scramble (negative control) in CRC and NC tissues in Cohort 3. **(f)** Multivariate cox regression analysis of lncSLCC1 were performed in TCGA dataset.

**
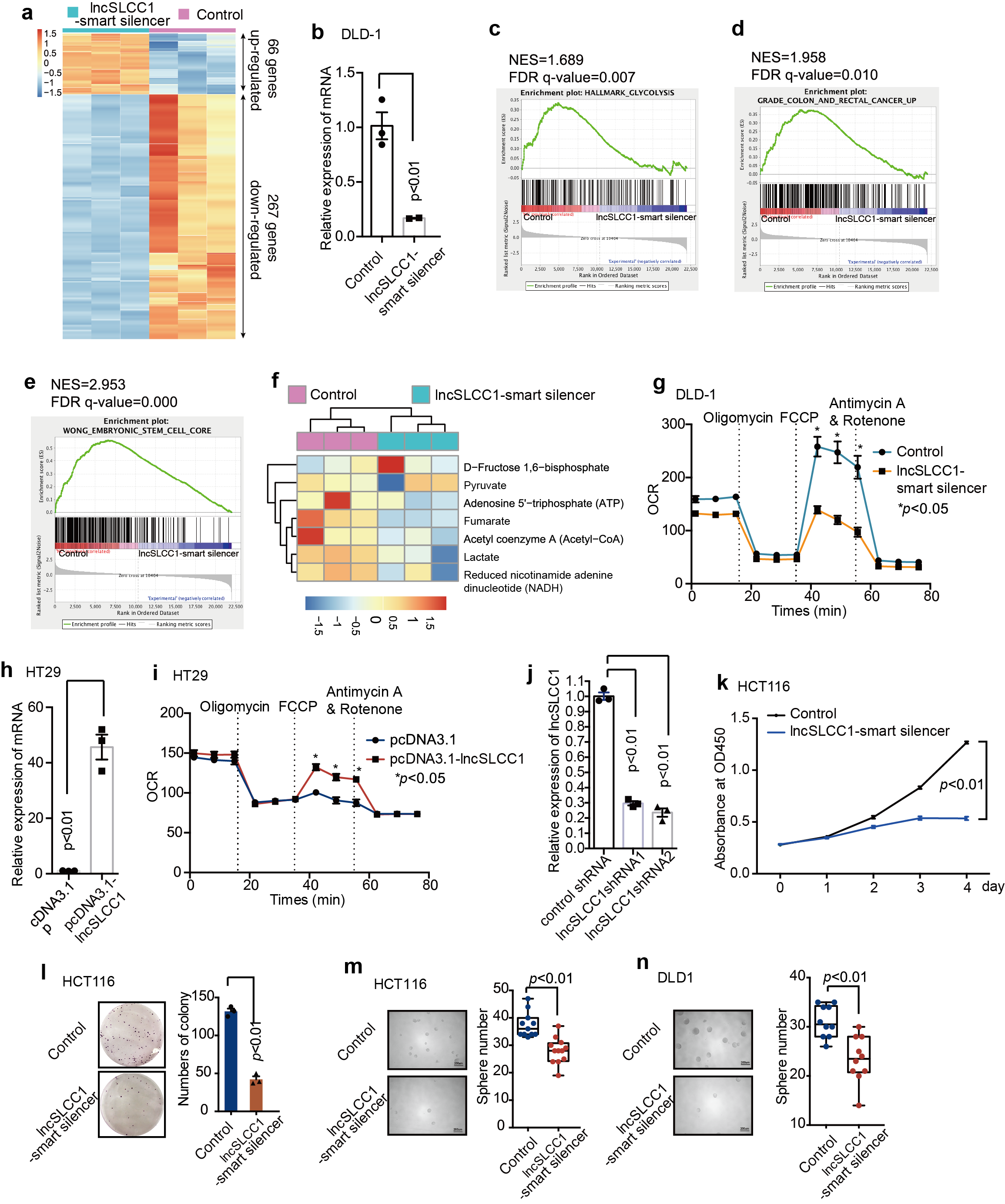
**

**Supplementary Fig. S3. LncSLCC1 activates CRC proliferation by driving glycolytic metabolism. (a**) The knockdown efficiency of lncSLCC1-smart silencer was confirmed in DLD-1 cells. **(b)** The heat map of genes differentially expressed in control and lncSLCC1 downregulated DLD-1 cells. **(c-e)** The GSEA analysis was conducted to identify the differential gene profiles between DLD-1 cells transfected with control or lncSLCC1-smart silencer. **(f)** LC-MS/MS-based analysis showed the major metabolites altered in the glucose metabolism in DLD-1 cells transfected with control and lncSLCC1-smart silencer. **(g)** OCR was detected in DLD-1 cells transfected with control or lncSLCC1-smart silencer, nonparametric Mann–Whitney test. **(h)** The overexpression efficiency of lncSLCC1 plasmid was confirmed in HT29 cells. **(i)** OCR was detected in HT29 cells transfected with control or lncSLCC1 overexpression plasmid, nonparametric Mann–Whitney test. **(j)** The knockdown efficiency of lncSLCC1-shRNA was confirmed. **(k)** Cell proliferation of HCT116 cells was measured by CCK8 assay after transfected with control or lncSLCC1-smart silencer , n=6, nonparametric Mann–Whitney test. **(l)** Colony formation assay was performed in HCT116 cells after transfection with control or lncSLCC1-smart silencer, n=3, nonparametric Mann–Whitney test. **(m-n)** Sphere assay was performed in HCT116 (m) and DLD-1 (n) cells after transfected with control or lncSLCC1-smart silencer , n=6, nonparametric Mann–Whitney test.

**
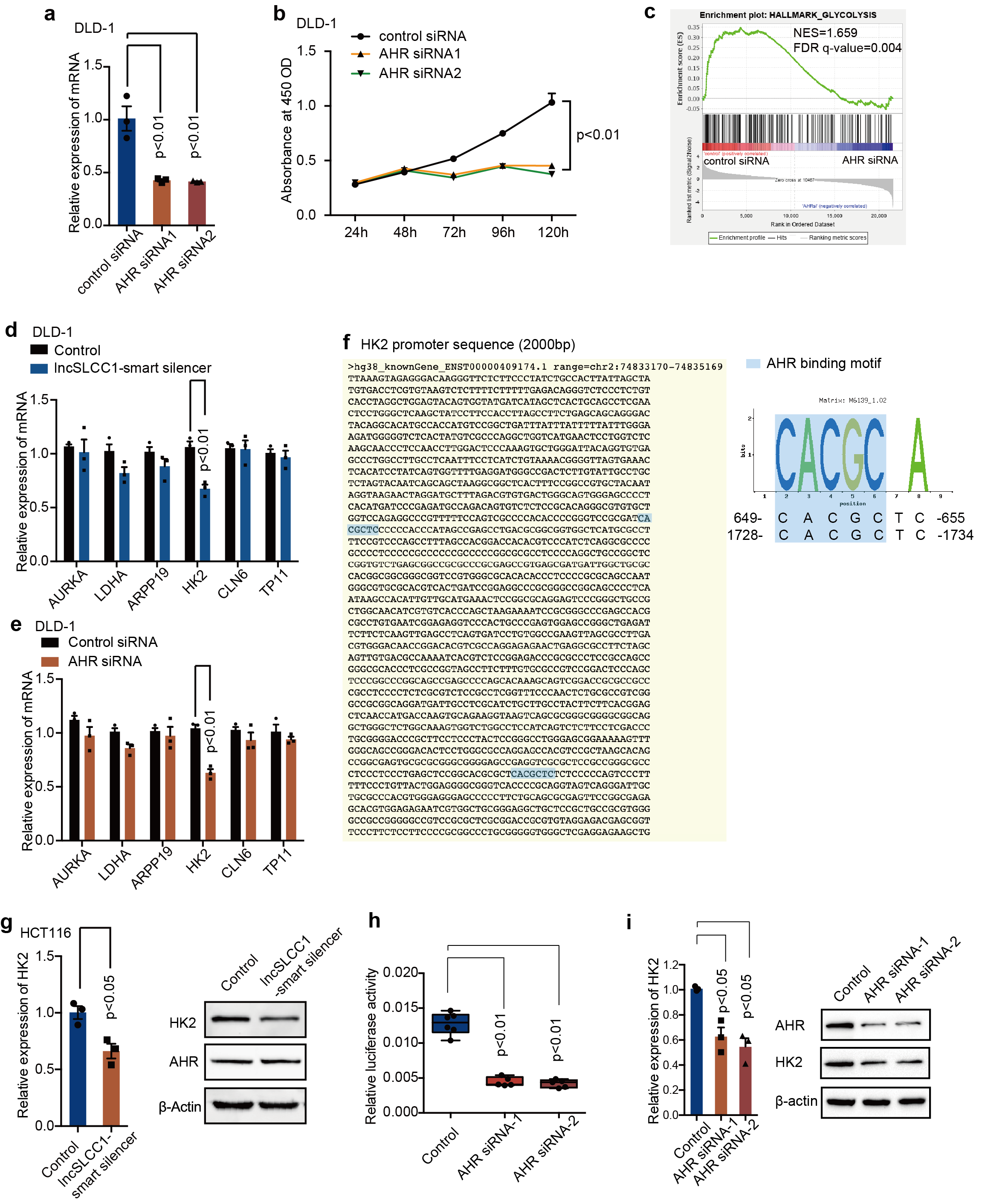
**

**Supplementary Fig. S4. LncSLCC1 interacts with AHR and regulates HK2 expression. (a)** The knockdown efficiency of AHR siRNA was confirmed in DLD-1 cells. **(b)** Cell proliferation of DLD-1 cells was measured by CCK8 assay after transfected with control siRNA or AHR siRNA1/2, n=6, nonparametric Mann–Whitney test. **(c)** The GSEA analysis was conducted to identify the differential gene profiles between DLD-1 cells transfected with control or AHR siRNA. **(d-e)** The mRNA level of target genes in DLD-1 cells transfected with control, lncSLCC1-smart silencer or AHR siRNA. **(f)** The predicted binding sites of AHR on the promoter sequence of HK2 from UCSC database. **(g)** The mRNA and protein level of HK2 in HCT116 cells transfected with control or lncSLCC1-smart silencer. **(h)** The HK2 promoter reporter vector was cotransfected with control siRNA or AHR siRNA into cells. Cells were harvested for luciferase activity assay. ANOVA. **(i)** The mRNA and protein level of genes in cells transfected with control or AHR siRNA.

**
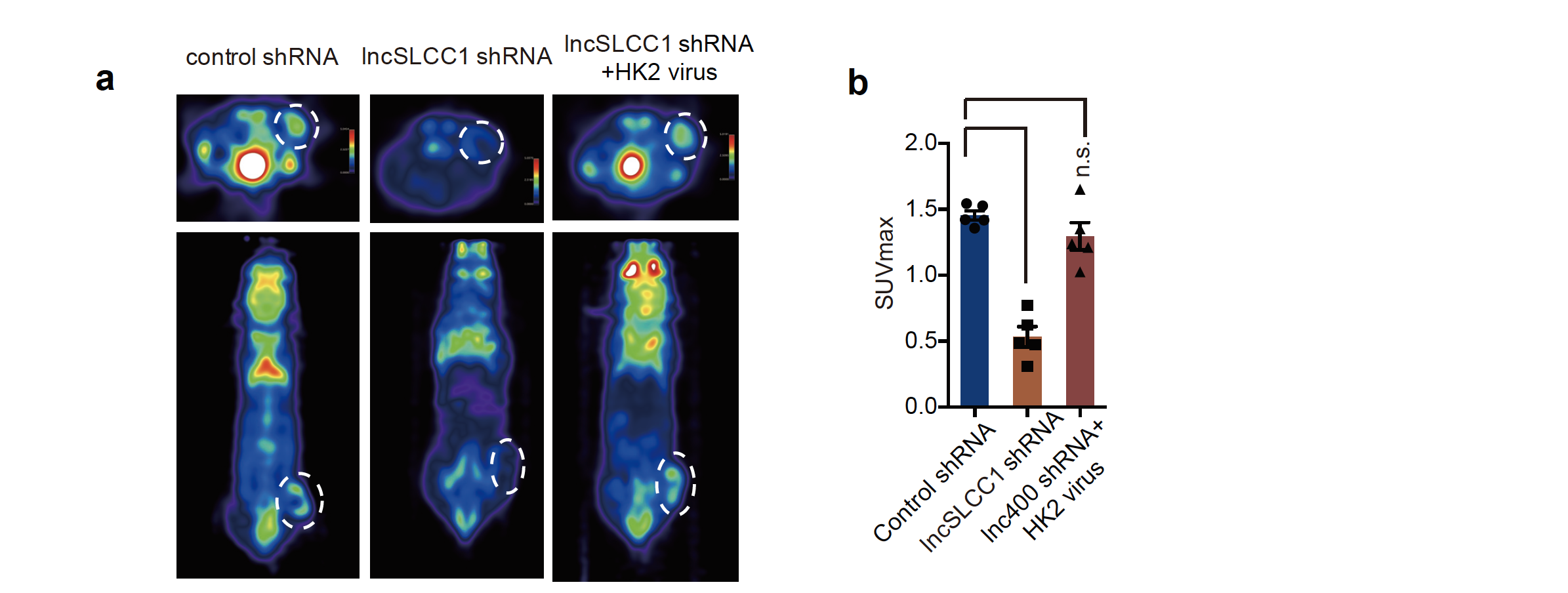
**

**Supplementary Fig. S5.** **HK2 is the functional target gene of lncSLCC1 in colorectal cancer.** (**a-b**) Representative images(**a**) and summarized data(**b**) of 18F-FDG uptake by micro-PET imaging in control shRNA, lncSLCC1 shRNA and lncSLCC1 shRNA with HK2 virus in xenograft mouse models (inoculated in the groin of mice, pictures were taken in the layer which showed the largest diameter of tumor and it made the non-tumor regions not in the same level). White circles indicated tumor glucose uptake. Maximum uptake values (SUVmax) for xenografts measured by FDG-PET were presented, nonparametric Mann–Whitney test.

**Supplementary Data**

**Supplementary Table S1.** The clinical information of colorectal cancer patients in Cohort3.

**Supplementary Table S2.** RNA-seq analysis in DLD-1 cells transfected with control siRNA or lncSLCC1 siRNA.

**Supplementary Table S3.** Proteins bands specific to lncSLCC1 identified by Mass Spectrometry (LC-MS) analysis.

**Supplementary Table S4.** RNA-seq analysis in DLD-1 cells transfected with control siRNA or AHR siRNA.

**Supplementary Table S5.** The sequences of siRNAs, primers and probe used in this study.
